# Damage Characterization of GFRP Hollow Ribbed Emergency Pipes Subjected to Low-Velocity Impact by Experimental and Numerical Analysis

**DOI:** 10.3390/polym16223116

**Published:** 2024-11-07

**Authors:** Ming Cheng, Dongdong Ding, Yaojun Ma, Sirong Zhu

**Affiliations:** 1 Hubei Key Laboratory of Mechanics Theory and Application of New Materials, Wuhan University of Technology, Wuhan 430070, China; 333570@whut.edu.cn; 2 School of Physics and Mechanics, Wuhan University of Technology, Wuhan 430070, China; 3 Hangzhou Tanko Machinery Technology Co., Ltd., Hangzhou 311400, China; dongdongding@163.com (D.D.); 13732209696@163.com (Y.M.)

**Keywords:** low-velocity impact, GFRP hollow ribbed emergency pipes, dynamic response, progressive damage, delamination

## Abstract

This paper investigates the low-velocity impact response and damage behavior of glass fiber reinforced polymer (GFRP) hollow ribbed emergency pipes of our design under different impact heights. Drop hammer impact tests with impact velocities of 8.41 m/s, 8.97 m/s, and 9.50 m/s were conducted using an impact platform. A progressive damage model for low-velocity impact was developed using Abaqus/Explicit finite element software. The model used the three-dimensional Hashin damage initiation criteria and a damage evolution model based on the equivalent strain method to simulate the initiation and evolution of intralaminar damage in the pipe ring. A cohesive zone model (CZM) based on a bilinear traction-separation law was used to simulate delamination. The results show that the pipe rings experienced fiber or matrix fractures and delamination damage during the impact process. Additionally, the pipe ring specimens underwent bending vibrations under the impact load, leading to fluctuating contact forces at all three impact heights. Analysis of the simulation results reveals that the primary damage modes in the GFRP hollow ribbed emergency pipe are fiber tension damage, matrix tension damage, and fiber compression damage, with delamination occurring mainly in the impact area and the interface area on both sides of the rib.

## 1. Introduction

Engineering safety issues are a major concern for society, as major safety accidents such as tunnel collapses and mine cave-ins often result in severe casualties and significant economic losses. Preventing injuries caused by falling debris during such collapses is an urgent issue that needs to be addressed in current tunnel and mine construction. In this scenario, deploying emergency pipes for temporary shelter and the evacuation of personnel is an effective solution [1,2,3,4]. Song et al. [5] investigated the dynamic response of capsule-encased composite metal shells under axial blast impacts within a tunnel environment. The experimental results indicated that the capsule-shaped composite shells exhibited no significant deformation after exposure to four blasts of varying intensities. Zhai et al. [6] designed a novel lifeboat capsule and examined its thermodynamic response under gas explosion loads, with results showing that the overall structure and key components met the design strength requirements. Mishra et al. [7] described the influence of impact loads on the deformation behavior of rock tunnel structures. Although these metal shell structures exhibit excellent deformation resistance, their substantial weight makes them difficult to transport.

Glass fiber reinforced plastic (GFRP), as a lightweight material, offers advantages such as low weight, high specific strength, impact resistance, and design flexibility, and GFRPs are widely used in fields such as the shipbuilding, aerospace, and automotive industries [8,9,10,11,12]. Compared to traditional metal materials, GFRP composites have a relatively lower modulus. In the current pipeline design, it is often necessary to increase the thickness of the structural layers to improve stiffness, but this leads to low structural efficiency (e.g., residual strength) and high manufacturing costs [13]. The introduction of hollow ribbed structures in composites effectively addresses this issue, achieving lightweight designs while maintaining load-bearing capacity and structural stiffness [14,15]. This study developed a GFRP hollow ribbed pipe using a continuous winding process, where the inner and outer walls resist normal stress and the circumferentially wound fiber-reinforced layer resists shear stress, significantly enhancing the tube’s circumferential stiffness. Additionally, the continuous winding process improves the interlayer mechanical properties, resulting in greater load-bearing capacity. Therefore, the application of GFRP hollow ribbed emergency pipes in emergency scenarios has become feasible.

However, due to the poor performance in the thickness direction, composite material structures such as GFRP emergency pipes are prone to delamination, matrix cracking, and fiber breakage when subjected to impacts in the thickness direction [16]. Barely visible impact damage (BVID), such as interlaminar delamination and back-face fiber breakage inside the pipe, is typically difficult to detect with the naked eye and is often overlooked in most applications. However, it is crucial, as it significantly affects the overall structural strength of the pipe [17]. Compared to metals with good ductility, the damage process in composite materials is more complex due to anisotropy and uneven stress distribution [18], especially for the novel GFRP hollow ribbed emergency pipe, whose impact response remains an urgent research topic. In this context, studying the damage behavior of hollow ribbed composite emergency pipes under impact loads is essential to understand the dynamic response of these pipes under low-velocity impacts, thereby providing a theoretical basis for their optimized design.

Currently, research on impact damage in composites mainly focuses on analyzing the low-velocity impact damage characteristics of solid laminated composite materials. Gemi et al. [19] studied the low-velocity impact response of (±55°)_3_ filament-wound E-glass/epoxy composite pipes under various impact energies and assessed the formation of impact damage. The results indicated that the damage progression in the pipe is influenced by radial displacement. Zhou et al. [20] developed a three-dimensional finite element model in Abaqus/Explicit based on an improved progressive damage model to study the dynamic mechanical response and damage progression of cross-ply laminated plates under low-velocity impact. The results showed that the proposed improvements are more suitable for low-velocity impact at various energy levels in composite laminates. Wu et al. [21] designed two stacking sequences for braided tubes and analyzed their low-velocity impact responses and damage behaviors. They further compared the quasi-static bending performance of intact and impacted tubes to assess the impact damage’s effect on residual performance. The results showed that the failure mode of impacted tubes is largely dependent on the outer braiding structure. Huang et al. [22] combined experimental and simulation methods to study the progressive damage of composite pipes with different stacking sequences, using a three-dimensional progressive damage model that considers nonlinear in-plane shear behavior to predict pipe failure. The results showed that their developed progressive damage model provided good predictions of failure mechanisms, ultimate strength, and load-deformation curves compared to experimental results. Khatounabadi et al. [23] conducted a low-velocity impact analysis on functionally graded porous circular plates reinforced with graphene sheets based on the first-order shear deformation theory, investigating the effects of various parameters on the contact force time history and central deflection of the plate. The results showed that the distribution of porosity has a greater impact on the contact force history and transient central deflection of the structure than other parameters. Many scholars have studied the damage characteristics of composites by combining experimental methods with finite element models based on the three-dimensional Hashin criterion to predict intralaminar damage [24,25,26]. The results indicated that the finite element simulation results are in good agreement with experimental results, validating the effectiveness of the finite element model. However, there is relatively limited research on the impact damage characteristics of hollow ribbed composite pipes using a combination of experimental and finite element analysis methods.

In this study, a drop-weight impact testing platform was designed and constructed to conduct impact tests with three different energy levels on GFRP hollow ribbed emergency pipes. A low-velocity impact progressive damage model was employed, in which the three-dimensional Hashin criterion was used to determine the onset of damage and a damage evolution model based on the equivalent strain method was utilized to simulate intralaminar damage progression. Additionally, a cohesive zone model based on a bilinear traction-separation law was used to simulate delamination in the pipe rings. By analyzing the impact response and damage characteristics of the GFRP hollow ribbed emergency pipes, their suitability for use in emergency scenarios was evaluated. Combining experimental and simulation approaches, the differences in the impact responses of GFRP hollow ribbed emergency pipes under varying impact heights were studied. Based on the simulation results, the damage behavior of the pipe rings under low-velocity impacts was evaluated, providing a reference for the structural optimization of GFRP hollow ribbed emergency pipes.

## 2. Impact Testing of GFRP Hollow Ribbed Emergency Pipes

### 2.1. The Layup Structure and Mechanical Properties of Pipes

**Figure 1:**
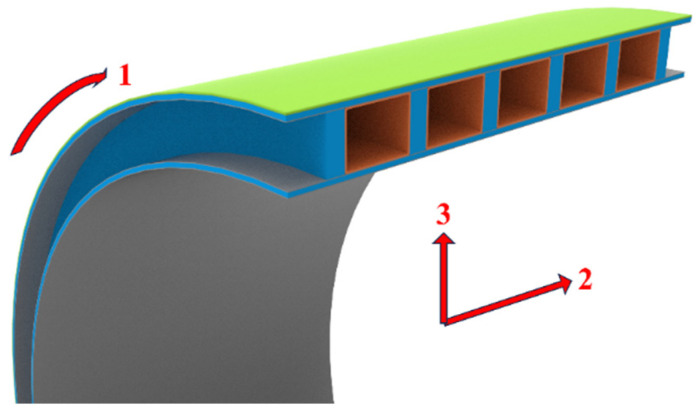
Cross-section of a GFRP hollow ribbed emergency pipe ring.

The structural layers of the GFRP hollow ribbed emergency pipe consist of inner and outer panels and a web of ribs. A cross-sectional schematic of the pipe ring specimen is shown in Figure 1, in which the circumferential direction is denoted as direction 1, the axial direction as direction 2, and the radial direction as direction 3. The orange section represents the polyethylene (PE) mold used for fabricating the ribs. The layup structure of the pipe ring is presented in Table 1, with the sequence of layers arranged from the inner side to the outer side of the pipe ring. The pipe rings were fabricated using 4800 tex glass fiber (386T), epoxy resin (4209A) as the matrix material, and an anhydride-based curing agent. All of the above materials were provided by Hangzhou Tanko Machinery Co., Ltd. (Hangzhou, China). A long pipe with an inner diameter of 800 mm was uniformly cut into pipe ring specimens with a length of 345 mm for experimental study. The mechanical properties of the layers in the pipe rings are provided in Table 2.

**Figure 2:**
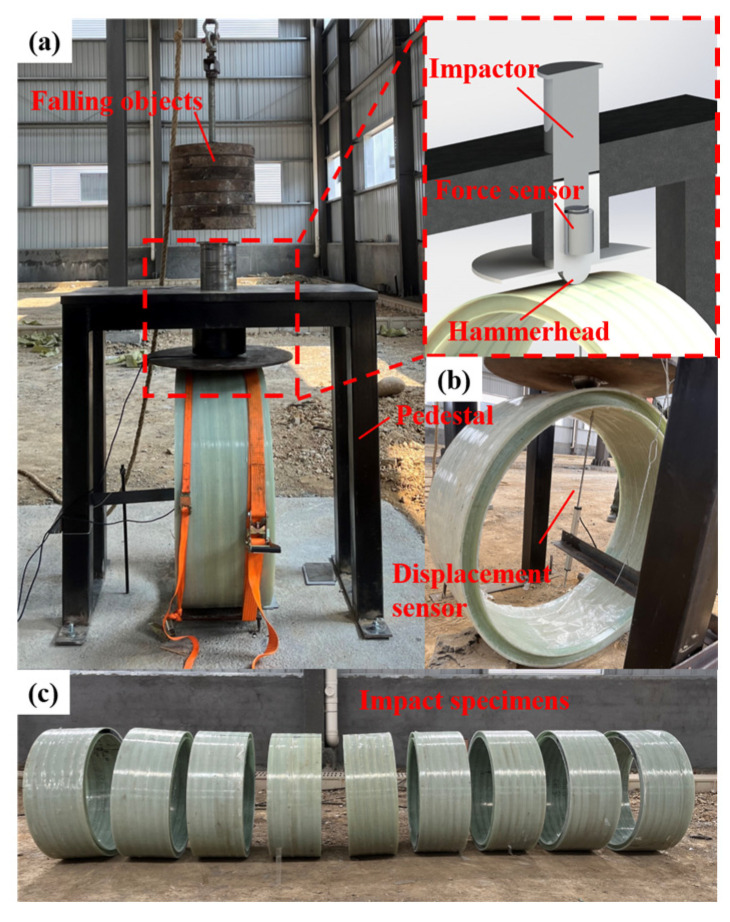
Schematic diagram of the impact platform: (**a**) overall; (**b**) bottom; (**c**) impact specimens.

The drop hammer impact platform primarily consists of a base, falling objects, an impactor, a hammerhead, and a traveling crane. Additionally, tension ropes are installed on the cement base to prevent the pipe ring from rebounding after the impact. The effective height for the drop-weight impact ranges from 2 m to 6 m, with the mass of the drop weight varying between 5 kg to 500 kg. The impact energy can be effectively altered by adjusting either the height of the drop or the mass of the drop weight. The D90-type hammerhead is used, and the displacement at the center point of the inner wall of the pipe ring is measured by a resistance-type displacement gauge (EY504-200, Jiangsu Yiyan Electronic Technology Co., Ltd., Jingjiang, China) with a range of 200 mm. The impact force is measured by a load sensor (YBY-500KN, Liyang Chaoyuan Instrument Factory, Liyang, China) with a range of 500 kN. To capture signals from the displacement and load sensors, a dynamic and static signal acquisition instrument (EY226, Jiangsu Yiyan Electronic Technology Co., Ltd., Jingjiang, China) was used for real-time data collection, set at a sampling frequency of 2 kHz and a sampling time of 0.5 ms. A manual trigger was used to control the sampling time range. The overall layout of the drop hammer impact platform and the measurement equipment are shown in Figure 2.

### 2.2. Progress of Impact Test

To simulate the scenario of falling rocks, a drop hammer impact platform was used to conduct drop hammer impact tests at three impact energies: 12.01 kJ, 13.68 kJ, and 15.32 kJ (corresponding to impact heights of 5 m, 5.5 m, and 6 m, respectively). Three impact tests were conducted for each energy level. The pipe ring specimen was placed in the center of the impact device and secured with tension straps. The hammerhead and impactor were positioned above the pipe ring specimen using the base’s sliding groove, ensuring that the hammerhead was directly above the hollow section of the pipe ring specimen. The falling objects were raised to the corresponding impact height and then released to conduct the impact test. The test was terminated when the pipe ring specimen came to rest on the ground and the radial displacement of the pipe ring, as recorded by the acquisition instrument, ceased to fluctuate.

## 3. Low-Velocity Impact Simulation Model

### 3.1. Damage Constitutive Model

Once the damage initiation criteria are met for the fibers and matrix, the material enters a damaged state. In this model, a damage coefficient is introduced based on Continuum Damage Mechanics (CDM) to calculate the degradation of the stiffness matrix, The relationship between the effective stress ($\hat{\sigma }$) and the nominal stress (*σ*) for a damaged composite pipe ring can be described as follows:

(1) $$\hat{\sigma }=d\cdot \sigma$$

In the equation, d represents the damage coefficient.

The constitutive equation for damaged states is [27]:

(2) $$\sigma_{ij}=C_{ij}^{d}\cdot \varepsilon_{ij}$$

In the formula, $C_{ij}^{d}$ represents the damaged orthotropic stiffness matrix. The stress-strain relationship based on this is as follows [28]:

(3) $$\left\{\begin{array}{c} \sigma_{11}\\ \sigma_{22}\\ \sigma_{33}\\ \sigma_{12}\\ \sigma_{23}\\ \sigma_{31} \end{array}\right\}=\left[\begin{array}{cccccc} C_{11}^{d} & C_{12}^{d} & C_{13}^{d} & 0 & 0 & 0\\ C_{12}^{d} & C_{22}^{d} & C_{23}^{d} & 0 & 0 & 0\\ C_{13}^{d} & C_{23}^{d} & C_{33}^{d} & 0 & 0 & 0\\ 0 & 0 & 0 & G_{12}^{d} & 0 & 0\\ 0 & 0 & 0 & 0 & G_{23}^{d} & 0\\ 0 & 0 & 0 & 0 & 0 & G_{31}^{d} \end{array}\right]\left\{\begin{array}{c} \varepsilon_{11}\\ \varepsilon_{22}\\ \varepsilon_{33}\\ {2\varepsilon }_{12}\\ {2\varepsilon }_{23}\\ {2\varepsilon }_{31} \end{array}\right\}$$

The stiffness coefficient ($C_{ij}^{d}$,$G_{ij}^{d}$) with damage is [29]:

(4) $$\left\{\begin{array}{l} C_{11}^{d}=\left(1-d_{f}\right)E_{11}\left(1-\nu_{23}\nu_{32}\right)\lambda ,\;\;\\ C_{22}^{d}=\left(1-d_{f}\right)\left(1-d_{m}\right)E_{22}\left(1-\nu_{13}\nu_{31}\right)\lambda ,\\ C_{33}^{d}=\left(1-d_{f}\right)\left(1-d_{m}\right)E_{33}\left(1-\nu_{12}\nu_{21}\right)\lambda ,\\ C_{12}^{d}=\left(1-d_{f}\right){\left(1-d_{m}\right)E}_{11}\left(\nu_{21}+\nu_{31}\nu_{23}\right)\lambda ,\\ C_{23}^{d}=\left(1-d_{f}\right){\left(1-d_{m}\right)E}_{22}\left(\nu_{32}+\nu_{12}\nu_{31}\right)\lambda ,\\ C_{13}^{d}=\left(1-d_{f}\right)\left(1-d_{m}\right)E_{11}\left(\nu_{31}+\nu_{21}\nu_{32}\right)\lambda ,\\ G_{12}^{d}=\left(1-d_{f}\right)\left(1-S_{mt}d_{mt}\right)\left(1-S_{mc}d_{mc}\right)G_{12},\\ G_{23}^{d}=\left(1-d_{f}\right)\left(1-S_{mt}d_{mt}\right)\left(1-S_{mc}d_{mc}\right)G_{23},\\ G_{31}^{d}=\left(1-d_{f}\right)\left(1-S_{mt}d_{mt}\right)\left(1-S_{mc}d_{mc}\right)G_{31} \end{array}\right.$$

The damage variable and λ are defined by the following equation [30]:

(5) $$d_{f}=1-\left(1-d_{ft}\right)\left(1-d_{fc}\right)\;d_{m}=1-\left(1-d_{mt}\right)\left(1-d_{mc}\right)\;\lambda =1/(1-\nu_{12}\nu_{21}-\nu_{23}\nu_{32}-\nu_{31}\nu_{13}-2\nu_{21}\nu_{32}\nu_{13})$$

In the equations, the subscripts *f* and *m* in the damage variables $d_{ft}$, $d_{fc}$, $d_{mt}$, and $d_{mc}$ represent the fiber and matrix modes, respectively, and the subscripts *t* and *c* represent the tensile and compressive modes. In defining the shear term $G_{ij}^{d}$, the factors $S_{mt}$ and $S_{mc}$ are introduced to control the reduction of shear stiffness due to tensile and compressive failures of the matrix, set at 0.95 and 0.5 in this model.

### 3.2. Damage Initiation Criterion

In this paper, the three-dimensional Hashin criterion based on the quadratic stress function [31] is used as a criterion for determining the onset of damage. Ait Mohammed et al. [28] compared numerical and experimental results under different impact energies, showing that the intralaminar damage model using the three-dimensional Hashin criterion has minimal error. The three-dimensional Hashin criterion is expressed as follows:

- Fiber tension ($\sigma_{11}\geq 0$):
  (6) $$F_{ft}={\left(\frac{\sigma_{11}}{X_{T}}\right)}^{2}+{\left(\frac{\sigma_{12}}{S_{12}}\right)}^{2}+{\left(\frac{\sigma_{13}}{S_{13}}\right)}^{2}\geq 1$$
- Fiber compression ($\sigma_{11}<0$):
  (7) $$F_{fc}={\left(\frac{\sigma_{11}}{X_{C}}\right)}^{2}\geq 1$$
- Matrix tension ($\sigma_{22}+\sigma_{33}\geq 0$):
  (8) $$F_{mt}={\left(\frac{\sigma_{22}+\sigma_{33}}{Y_{T}}\right)}^{2}+\frac{1}{S_{23}^{2}}\left(\sigma_{23}^{2}-\sigma_{22}\sigma_{33}\right)+{\left(\frac{\sigma_{12}}{S_{12}}\right)}^{2}+{\left(\frac{\sigma_{13}}{S_{13}}\right)}^{2}\geq 1$$
- Matrix compression ($\sigma_{22}+\sigma_{33}<0$):
  (9) $$F_{mc}={\left(\frac{\sigma_{22}+\sigma_{33}}{2S_{23}}\right)}^{2}+\frac{\sigma_{22}+\sigma_{33}}{Y_{C}}\left[{\left(\frac{Y_{C}}{2S_{23}}\right)}^{2}-1\right]+\frac{1}{S_{23}^{2}}\left(\sigma_{23}^{2}-\sigma_{22}\sigma_{33}\right)+{\left(\frac{\sigma_{12}}{S_{12}}\right)}^{2}+{\left(\frac{\sigma_{13}}{S_{13}}\right)}^{2}\geq 1$$

In the formula, $X_{T}$, $X_{C}$, $Y_{T}$, $Y_{C}$, and $S_{12}$ represent the longitudinal tensile strength, longitudinal compressive strength, transverse tensile strength, transverse compressive strength, and in-plane shear strength, respectively, while $S_{13}$ and $S_{23}$ represent the transverse shear strength.

### 3.3. Damage Evolution

**Figure 3:**
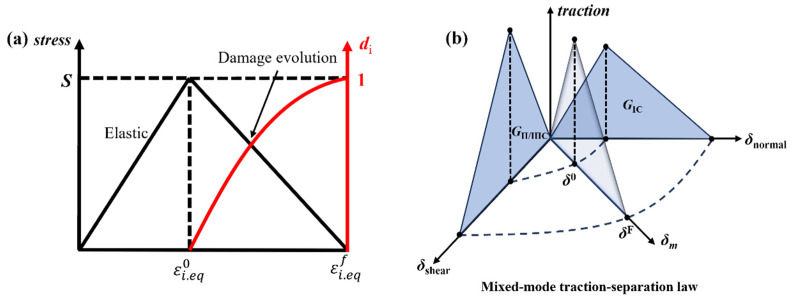
Bilinear model: (**a**) damage evolution model; (**b**) cohesive zone model.

After satisfying the damage initiation criterion, continued loading will lead to a reduction in the stiffness of the composite material emergency pipe ring. Therefore, using an appropriate damage evolution model effectively simulates the expansion of intralaminar damage. In this paper, a linear damage evolution model based on the equivalent strain method [32] is used, as shown in Figure 3a. By introducing the characteristic length of the element, this model reduces its dependence on mesh density. The damage variables in the model are determined by the relationship between equivalent strain and fracture energy, and the damage variables for both fibers and matrix are defined in the following form:

(10) $$d_{i}=\frac{\varepsilon_{i,eq}^{f}\left(\varepsilon -\varepsilon_{i,eq}^{0}\right)}{\varepsilon \left(\varepsilon_{i,eq}^{f}-\varepsilon_{i,eq}^{0}\right)}(\varepsilon_{i,eq}^{0}\leq \varepsilon \leq \varepsilon_{i,eq}^{f},i=ft,fc,mt,mc)$$

(11) $$\varepsilon_{i,eq}^{0}=\frac{X_{i}}{E_{0,j}}{,\varepsilon }_{i,eq}^{f}=\frac{2G_{i}}{l_{e}X_{i}}$$

where $\varepsilon_{i,eq}^{f}$ is the equivalent destructive strain on the fiber or matrix at fracture and $\varepsilon_{i,eq}^{0}$ is the equivalent strain on the fiber or matrix at the time of initial damage. $X_{i}$ represents the tensile and compressive strengths of the fiber and the tensile and compressive strengths of the matrix. $E_{0,j}$ denotes the initial elastic modulus in the direction of fibers and matrix. $G_{i}$ represents the fracture energy for the four damage modes. The term $l_{e}$ denotes the characteristic length of the element, which is the square root of the element volume divided by the layer thickness.

### 3.4. Interlaminar Damage Model

In this paper, cohesive elements based on the bilinear traction-separation law are inserted to simulate delamination damage sustained by composite emergency pipe rings. The cohesive zone model can effectively predict the initiation and evolution of interlaminar delamination damage, as shown in Figure 3b. The quadratic stress criterion proposed by Camanho [33] is used to determine the initiation of delamination damage and the energy-based B–K criterion by Benzeggagh and Kenane [34] serves as the criterion for damage evolution. The expressions are as follows:

(12) $${\left(\frac{\langle t_{n}\rangle }{t_{n}^{0}}\right)}^{2}+{\left(\frac{t_{s}}{t_{s}^{0}}\right)}^{2}+{\left(\frac{t_{t}}{t_{t}^{0}}\right)}^{2}=1$$

In the formulas, $t_{n}$, $t_{s}$, and $t_{t}$ represent the nominal traction stresses for Mode I (opening), Mode II (sliding), and Mode III (tearing), respectively. The terms $t_{n}^{0}$, $t_{s}^{0}$, and$\;t_{t}^{0}$ denote the interfacial strengths in the three principal directions, and 〈 〉 represents the Macaulay operator. The B–K criterion, used to simulate the evolution of interlaminar delamination damage, is expressed as follows:

(13) $$G_{C}=G_{IC}+\left(G_{IIC}-G_{IC}\right){\left(\frac{G_{S}}{G_{T}}\right)}^{\eta }$$

In the expression, $G_{C}$ represents the fracture toughness under mixed-mode conditions; $G_{S}=G_{II}+G_{III}$ and $G_{T}=G_{S}+G_{I}$, where $G_{S}$ and $G_{T}$ are the tangential and total strain energy release rates, respectively; $G_{IC}$ and $G_{IIC}$ are the critical fracture energies for the normal and shear modes, respectively; and η is the coefficient for the B-K criterion.

### 3.5. Finite Element Model

**Figure 4:**
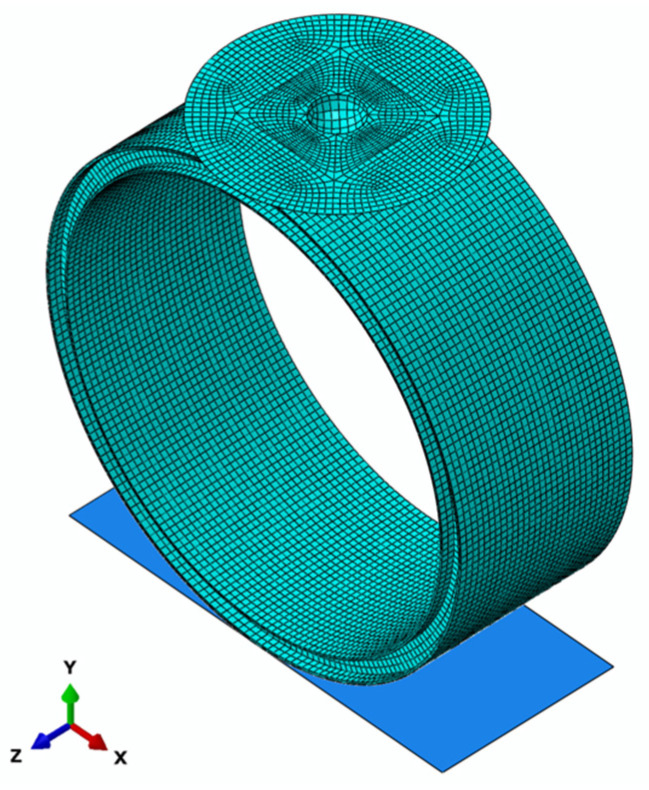
Impact finite element model of GFRP ribbed emergency pipe.

Impact simulations were performed using the Abaqus/Explicit 2021 finite element software. To enhance computational efficiency, a quarter-model of the pipe ring and hammerhead [35] was created based on symmetry. Symmetric boundary conditions were applied to the pipe ring. In the quarter model, the plane perpendicular to the pipe’s axial direction (*z*-axis) had the boundary conditions U3 = UR1 = UR2 = 0, while the plane perpendicular to the radial direction (*x*-axis) had boundary conditions U1 = UR2 = UR3 = 0, where 1, 2, and 3 represent the *x*-, *y*-, and *z*-axes, respectively. To match the experimental conditions, all degrees of freedom of the ground were constrained, while all degrees of freedom of the hammer except for vertical displacement were constrained. The pipe ring and PE mold were meshed using 8-node linear reduced integration solid elements (C3D8R), with a global mesh size of 12 mm × 12 mm. The hammerhead is modeled as a rigid body using R3D4 elements. Zero-thickness cohesive elements (COH3D8) are inserted between different laminates to simulate interfaces. Table 3 lists the intralaminar fracture energy parameters and interlaminar interface parameters for the GFRP hollow-core reinforced emergency pipes. Impact velocity is applied as an initial condition at the reference point of the hammerhead, with impact velocities corresponding to three impact heights being 8.41 m/s, 8.97 m/s, and 9.50 m/s. Mass is applied to the reference point of the hammerhead in the interaction inertia options. Contact between the hammerhead and the pipe ring, as well as between the pipe ring and the ground, is defined using a general contact method based on a penalty contact algorithm to prevent node interpenetration, with a penalty factor set to 0.2 in this study. The intralaminar progressive damage model and the interlaminar damage model are implemented through the user-defined material subroutine (VUMAT) in Abaqus/Explicit, with damage to the PE mold not considered. The finite element model is shown in Figure 4.

## 4. Results and Discussion

### 4.1. Model Validation and Low-Velocity Impact Response

**Figure 5:**
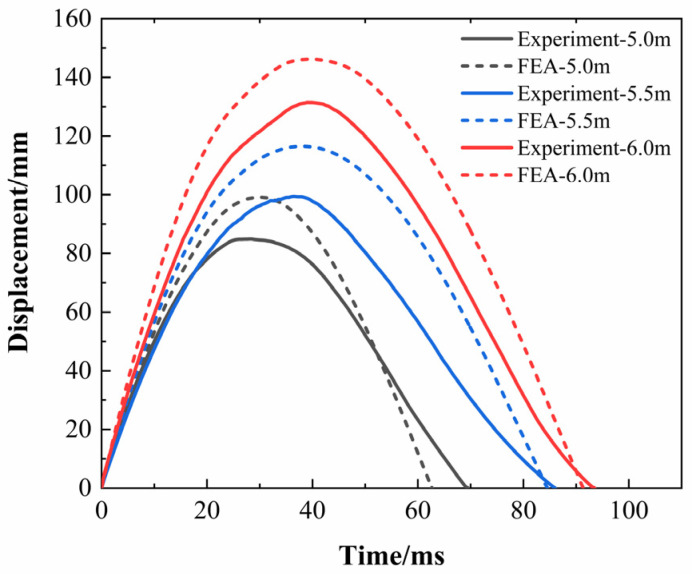
Displacement–time curve.

Table 4 presents the experimental results for the maximum displacement and maximum impact force. Figure 5 shows the displacement–time comparison curves of the center point of the inner wall of the pipe rings under three impact heights. The maximum displacements at the center point of the inner wall of the pipe ring under three impact heights were 84.9 mm, 99.46 mm, and 131.49 mm, respectively. The corresponding results of numerical calculation were 99.14 mm, 113.20 mm, and 146.21 mm, respectively. The resulting errors were 16.77%, 13.82%, and 11.20%, respectively, indicating that the calculation results of the finite element model were in good agreement with the experimental results. The three displacement-time comparison curves indicate that the simulated displacement values are higher than the experimental values, and the structure exhibits lower stiffness. This is because, in numerical simulations, the failed elements are deleted, whereas in reality the failed structure still exists [28]. Additionally, the inner diameters of the pipe ring specimens have returned to their original state after being subjected to impact loads, ensuring smooth passage for personnel. This indicates that the pipe rings meet the design requirements.

**Figure 6:**
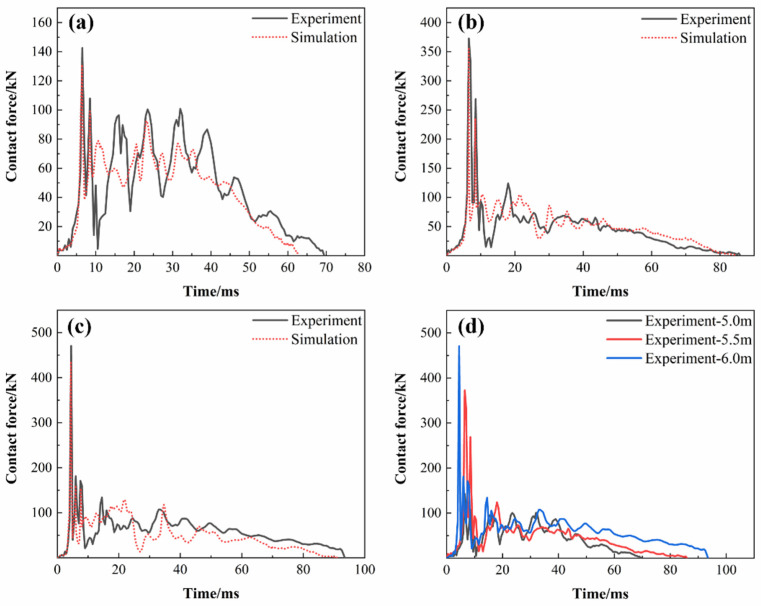
Contact force–time curves: (**a**) 5 m impact height; (**b**) 5.5 m impact height; (**c**) 6 m impact height; (**d**) Comparison of experiments.

Figure 6 shows the contact force–time comparison curves between the hammerhead and the pipe ring obtained from impact tests and numerical calculations at three different heights. The numerical calculations show a trend in contact force similar to that observed in the experiments. When the hammerhead initially contacts the pipe ring, the contact force fluctuates and rises due to the preliminary fiber breakage and matrix cracking at the contact surface, coupled with the bending vibration of the pipe ring under the impact load [37]. As the contact area increases, the contact force quickly reaches a peak when the surface of the circular steel plate attached to the hammerhead fully contacts the pipe ring surface. The contact force then decreases to a certain value after the impactor rebounds, a phenomenon observed in all three impact height curves. As the impactor and hammerhead collide again, the contact force reaches another peak. With further impacts, part of the hammerhead’s impact energy is converted into internal energy within the pipe ring, while the rest is dissipated through collisions between the impactor and hammerhead and damage to the pipe ring during impact, until the hammerhead’s impact energy is fully dissipated. Subsequently, the pipe ring’s elastic potential energy is released, causing the hammerhead to rebound. During the separation of the hammerhead from the pipe ring specimen, the contact force decreases along with the intense vibration of the pipe ring, eventually dropping to zero once the hammerhead fully detaches from the pipe ring. Figure 6d compares the contact force variations at the three impact heights, revealing that the peak contact force occurs later as the impact height decreases. Higher impact energy enables the pipe ring structure to exhibit a faster impact response. The contact force–time curve results presented in this paper do not exhibit an irregular sine wave shape as reported by Gemi et al. [38], which is because the impactor and hammerhead in the test equipment are not mechanically fixed together. There is relative movement between the impactor and hammerhead during impact, resulting in multiple impacts, which closely resembles the real-world usage conditions of the emergency pipe.

### 4.2. Analysis of Impact Damage Behavior

**Figure 7:**
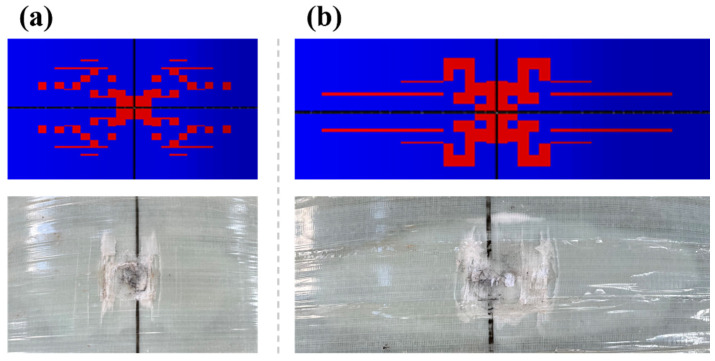
Comparison of damage results of impact points in simulation and experiment: (**a**) 5 m impact height; (**b**) 6 m impact height.

For intralaminar damage, which includes fiber tension/compression damage and matrix tension/compression damage, the state variables used in this finite element analysis to assess the damage conditions are the fiber tension damage state variable (SDV1), fiber compression damage state variable (SDV2), matrix tension damage state variable (SDV3), matrix compression damage state variable (SDV4), and total damage state variable (SDV5). Figure 7 compares the total damage state at the impact point of the pipe ring for the impact heights of 5 m and 6 m with the final damage patterns observed in the experiments. Red indicates complete failure. The results show that no penetration occurred in the pipe ring samples at either height. Higher impact heights caused larger surface damage areas. The high shear stress at the contact edge between the pipe ring surface and the hammerhead, coupled with tensile stress generated by compression on the unidirectional fabric, caused the fibers and resin at the contact edge to exhibit a “whitening” phenomenon once the tensile and shear strength of the unidirectional fabric were exceeded. Observing the overall appearance of the pipe ring, under an impact load from a 5 m drop height, the final damage pattern at the impact point resembles a peanut shape. This is the combined result of interlayer delamination and intralayer damage, consistent with the findings reported by Gemi et al. [38]. As the impact height increases, the damage morphology at the impact point changes to an irregular elliptical shape, with the major axis of the ellipse aligned with the fiber direction of the circumferential wrap layers. This shape transition occurs because the propagation speed of impact stress waves along the fiber direction is faster than in other directions, and this effect becomes more pronounced with higher impact energy [39].

**Figure 8:**
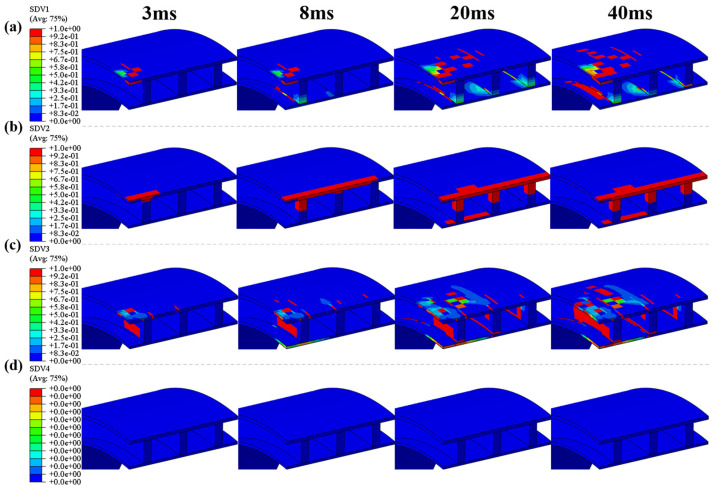
Progressive damage within the top layer of pipe rings at different times at impact height 5 m: (**a**) fiber tension damage; (**b**) fiber compression damage; (**c**) matrix tension damage; (**d**) matrix compression damage.

**Figure 9:**
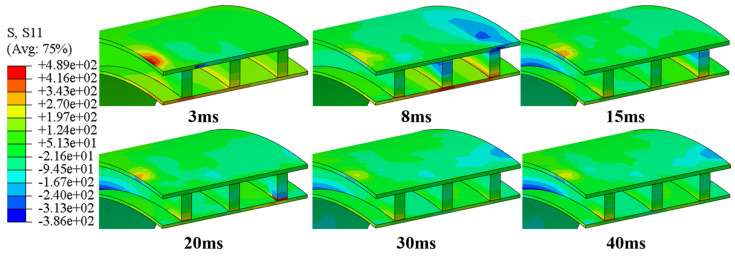
Circumferential stress transfer process at the impact point at the impact height of 5 m.

**Figure 10:**
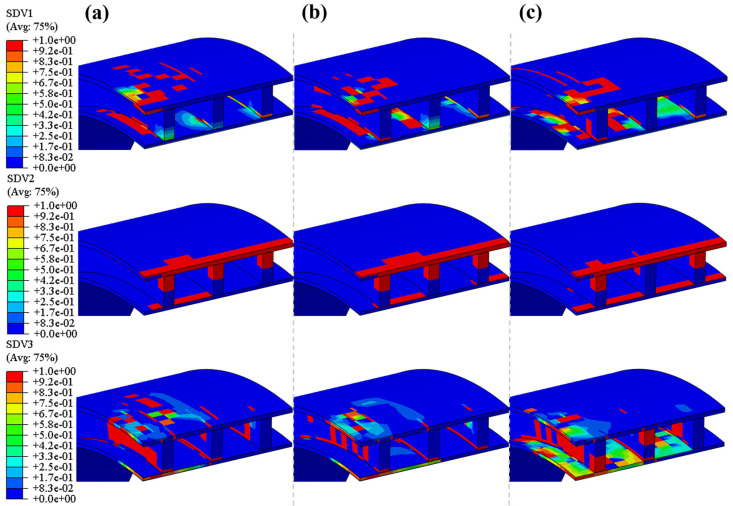
Final progressive damage: (**a**) 5 m impact height; (**b**) 5.5 m impact height; (**c**) 6 m impact height.

Figure 8 shows the changes in damage patterns at different times during the impact process at a height of 5 m on the top (impact site) of a quarter of the pipe ring sample. The study finds that different parts of the emergency pipe exhibit different failure modes during the impact process. The results indicate that throughout the impact process, the main damage modes at the top of the emergency pipe (impact site) are fiber tension damage and matrix tension damage, with no matrix compression failure occurring. Figure 9 illustrates the variations of circumferential stress during the impact process. From the stress contour map, it is evident that at the initial impact stage, peak stress emerges around the impact point, while only minor stress appears directly at the impact point. This occurs because the fibers at the impact point undergo compressive failure due to instantaneous compression, leading to a sudden stress reduction. Meanwhile, high tensile stress is generated around the impact point due to contact between the impactor and the outer surface of the pipe, resulting in tensile failure in both the fibers and the matrix. Additionally, stress propagates from the impact zone through the upper to the lower layers of the pipe. The bending-induced tensile stress in the matrix of the reinforcing ribs near the impact center causes matrix tensile failure in the ribs, as corroborated by the damage contour results at 3 ms in Figure 8b. With continued impact, stress accumulates at the joint between the ribs and the inner solid wall, causing the fiber tensile damage zone to expand continuously. Vibrations during the impact process contribute to the discontinuity in the expansion of fiber tensile damage. Simultaneously, the reinforcing rib layer has greater stiffness compared to the outer solid wall. This stiffness discrepancy causes substantial compressive stress in the second reinforcing rib near the impact center, close to the outer solid wall, which transfers to the third rib, further extending the fiber compressive damage. Observing Figure 8c, it is apparent that only ply 2 exhibits matrix tensile failure within the inner solid wall, while adjacent plies 1 and 3 show no significant failure. Under identical deformation, layers with a higher modulus generate greater stress than those with a lower modulus. Ply 2, with a lower transverse tensile strength than the other two layers, fails more easily, corroborating this phenomenon. Additionally, matrix tension damage is most severe in the layers bonded with the reinforcements, primarily due to stress concentration and the stiffness imbalance. Additionally, the bending deformation increasingly expands from the inner to the outer layers, generating significant tensile stress. The transfer of interlaminar loads exacerbates matrix tension damage, causing the matrix tension damage area to progressively expand from the inner panel to the outer panel [24]. When the impact force first reaches its peak, the damage has not yet reached its most severe state, indicating that the damage accumulates gradually within the pipe ring. Figure 10 compares the final progressive damage states under three impact heights. From the figure, it can be observed that under all three impact heights, only a small area of fiber tensile failure appears in the reinforcing ribs of the pipe ring. This limited damage is due to the high longitudinal tensile and compressive strength, which provides the pipe ring with a substantial damage tolerance overall. Fiber compressive failure is limited to the region near the impact point along the axial direction of the pipe ring. As the impact height increases, the area of matrix tensile damage gradually expands on the inner wall surface of the pipe ring at locations corresponding to the ribs. Additionally, the damage area on the inner panel is larger than that on the outer panel, indicating that matrix tensile damage decreases progressively from the bottom layer to the top layer, consistent with the observations reported by Lou et al. [40].

**Figure 11:**
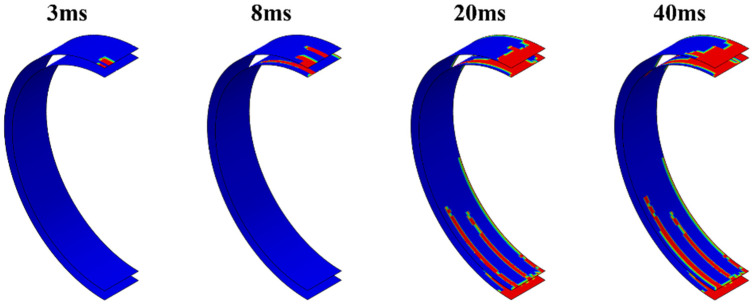
Damage evolution of the outer and inner interfaces of pipe rings at impact height 5 m.

**Figure 12:**
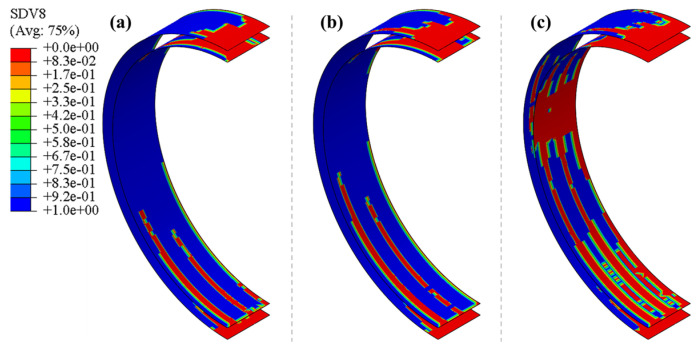
Final delamination: (**a**) 5 m impact height; (**b**) 5.5 m impact height, (**c**) 6 m impact height.

**Figure 13:**
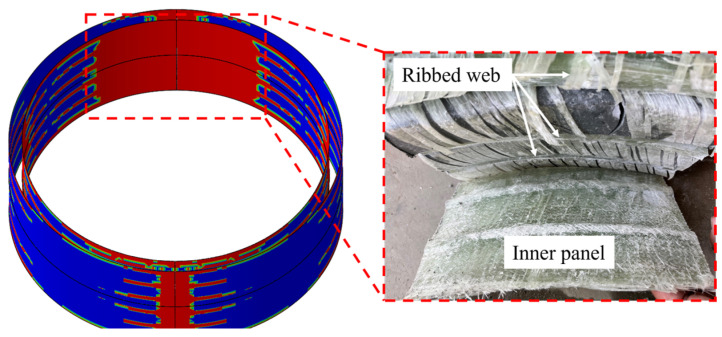
Comparison of delamination between experiment and simulation.

Due to the unconstrained edge positions of the reinforcements, the connection areas between the reinforcements and the inner and outer panels often experience higher bending stresses under impact loads, making these areas more susceptible to interface delamination. Therefore, this study focuses solely on the delamination at the interfaces between the reinforcements and the inner and outer panels. Figure 11 shows the evolution of damage at the outer and inner interfaces of the pipe ring at an impact height of 5 m. The red areas indicate complete failure, the blue areas represent undamaged regions, and the other colored areas denote partial damage. The results indicate that the partial damage areas surround the completely failed regions, suggesting that the delamination damage extends outward from the centers of the layers. At the onset of the impact, delamination occurs only at the impact site. As the impact progresses, the delaminated area on the inner side of the reinforcement becomes larger than that on the outer side. This is because the circumferential stiffness of the outer panel is greater than that of the inner panel, causing the bending neutral axis during the impact to shift toward the outer panel, resulting in higher shear stresses on the inner interface compared to the outer interface. Additionally, the delamination area spreads outward from the impact point’s center. After the circular steel plate connected to the hammerhead fully contacts the pipe ring, the delamination area extends downward along the circumferential direction of the pipe ring at the position corresponding to the ribs. Meanwhile, the delamination area at the bottom interface of the pipe ring extends upward along the circumferential direction at the position corresponding to the ribs. The shorter the distance to the impact center, the larger the delamination area, as the bending deformation of the pipe ring is greater closer to the impact point, generating larger tensile stresses in the interface layer. Figure 12 compares the final delamination conditions under three different impact heights. The results show that the interface delamination near the impact point of the tubular ring specimens is generally consistent across all three impact heights. Compared to the 5 m impact height, the higher impact energy at 5.5 m results in greater bending deformation, leading to an expansion of the delamination area at the interface. As the impact height further increases to 6 m, delamination occurs at the interface of the tubular ring’s waist region, which is a consequence of further increased bending deformation. With the increase in impact energy, the delamination area at the bottom of the tubular ring expands further. In addition, the delamination region of the interface on both sides of the rib expands with the increase of the impact height, while there is no obvious damage expansion pattern in the hollow part of the pipe ring, which is a result of the inconsistency between the stiffness of the rib and the hollow part. Figure 13 compares the delamination observed in the cut top segment of the pipe ring specimen from the experiment with the delamination damage predicted by the simulation. Partial delamination occurs at the interface between the reinforcements and the outer panel, indicating that the reinforcements and outer panel are not completely debonded. In contrast, complete delamination at the interface between the reinforcements and the inner panel results in a full debonding of the reinforcements from the inner panel. This comparison shows that the simulation predictions of delamination are in good agreement with the experimental results.

## 5. Conclusions

Low-velocity impact tests and finite element simulation were carried out on GFRP hollow ribbed emergency pipes of our design at different impact heights, and the impact response and damage characteristics during impact were studied. The methods and conclusions in this paper provide guidance for the optimal design of a GFRP hollow ribbed emergency pipe. The conclusions are as follows:

- The maximum dent deformation values for the GFRP hollow ribbed emergency pipes subjected to low-velocity impact loads of 9.02 kJ, 10.27 kJ, and 11.52 kJ were 84.9 mm, 99.46 mm, and 131.49 mm, respectively. The pipes were able to recover their initial state after rebound, indicating their reliability for use in emergencies. The impact tests conducted using the impact platform showed multiple peaks in contact force, and the contact force fluctuated throughout the impact process, which aligns with the actual conditions.
- The progressive damage model used in this study employs the three-dimensional Hashin damage initiation criterion to determine the onset of intralaminar damage and utilizes a damage evolution model based on the equivalent strain method to simulate the progression of intralaminar damage. This model effectively simulates the mechanical response of the emergency pipe ring under low-velocity impact. The errors between the simulated and experimental displacements at the center point of the pipe ring’s inner wall were 16.77%, 13.82%, and 11.20%, respectively.
- The final damage morphology at the impact point of the pipe ring approximates a peanut shape. As the impact height increases, the damage morphology at the impact point of the pipe ring evolves into an irregular elliptical shape. The primary damage modes at the top of the emergency pipe (the impact area) are fiber tensile damage and matrix tensile damage. At all three impact heights, the pipe ring’s reinforcing ribs exhibited only localized fiber tensile failure. After the impact, the pipe ring as a whole maintained a high level of damage tolerance.
- The delamination damage inside the pipe ring is more severe than that outside the pipe ring, and the delamination area spreads from the center of the impact point to the surroundings. Higher impact heights result in larger delamination areas. The propagation pattern of delamination damage is influenced not only by the load but also by the position of the rib. The comparison between simulation results and experiments verifies the validity of the application of the cohesive zone model.

## Figures and Tables

**Table 1:** The layup structure of the GFRP hollow ribbed emergency pipe ring.

Component	Layup (Color)	*t*/(mm)	Material
Inner panel	Ply1 (gray)	0.56	Knitted felt
	Ply2 (blue)	2.87	Glass fiber, 4800 tex
	Ply3 (gray)	0.56	Knitted felt
Ribbed web	Ply4 (blue)	35	Glass fiber, 4800 tex
Outer panel	Ply5 (gray)	0.56	Knitted felt
	Ply6 (blue)	2.99	Glass fiber, 4800 tex
	Ply7 (green)	1.41	Unidirectional fabric

Notes: *t*—Layer thickness; color—Corresponds to the color of each layer in Figure 1.

**Table 2:** Mechanical property parameters of GFRP hollow ribbed emergency pipe ring.

Layup	*E_ii_* [GPa]	$\nu_{12}$	*G_ij_* [GPa]	*X*_t_ [MPa]	*X*_c_ [MPa]	*Y*_t_ [MPa]	*Y*_c_ [MPa]	*S_ij_* [MPa]
Ply1/3/5	13.6/13.6/13.6	0.28	4.5/4.5/4.5	120	110	120	110	66
Ply2/4/6	41.9/9.2/9.2	0.29	2.9/2.9/2.9	452.5	362	40.1	48.1	36.5
Ply7	5.7/36.5/5.7	0.29	2.5/2.5/2.5	60	70	400	280	45
PE	1	0.3	-	-	-	-	-	-

Notes: *E_ii_* (*i* = 1, 2, 3)—Elastic modulus in direction *i*; *ν*_ij_ (*i* = 1, 2; *j* = 2, 3)—Poisson’s ratio in different directions; *G_ij_* (*i* = 1, 2; *j* = 2, 3)—Shear modulus in different directions, *X*_t_ and *X*_c_—Longitudinal tensile and compressive strengths; *Y*_t_ and *Y*_c_—Transverse tensile and compressive strengths; *S_ij_* (*i* = 1, 2, 3; *j* = 1, 2, 3)—shear strength.

**Table 3:** Intralaminar fracture energy parameters and interlaminar interface parameters [20,36].

Parameter	Value
Fiber fracture energy *G*_ft_	91.6 N·mm^−1^
Fiber fracture energy *G*_fc_	79.9 N·mm^−1^
Matrix fracture energy *G*_mt_	0.22 N·mm^−1^
Matrix fracture energy *G*_mc_	1.1 N·mm^−1^
Density *ρ*	1.0 g·cm^−3^
Interfacial penalty stiffness *K*_n_ = *K*_s_ = *K*_t_	1 × 10^6^ N·mm^−3^
Normal strength *T*_n_	30 MPa
Shear strength *T*_s_ = *T*_t_	53 MPa
Mode I fracture toughness *G*_IC_	1.19 mJ·m^−2^
Mode II/III fracture toughness *G*_IIC_ = *G*_IIIC_	2.0 mJ·m^−2^
Power factor *η*	1.8

**Table 4:** Experimental results at different impact energies.

Impact Energy (kJ)	Maximum Displacement (mm)	Average Value (mm)	Standard Deviation	Maximum Impact Force (kN)	Average Value (kN)	Standard Deviation
12.01	81.51	84.9	2.77	135.28	142.55	5.94
	84.9			142.55		
	88.29			149.82		
13.68	94.79	99.46	3.82	349.55	372.65	18.86
	99.46			372.65		
	104.13			395.75		
15.32	124.53	131.49	5.69	442.44	470.68	23.06
	131.49			470.68		
	138.46			498.92		

## Data Availability

The original contributions presented in the study are included in the article. Further inquiries can be directed to the corresponding author.
